# Effects of Solvent Additive and Micro-Patterned Substrate on the Properties of Thin Films Based on P3HT:PC70BM Blends Deposited by MAPLE

**DOI:** 10.3390/ma16010144

**Published:** 2022-12-23

**Authors:** Marcela Socol, Nicoleta Preda, Carmen Breazu, Gabriela Petre, Anca Stanculescu, Ionel Stavarache, Gianina Popescu-Pelin, Andrei Stochioiu, Gabriel Socol, Sorina Iftimie, Christine Thanner, Oana Rasoga

**Affiliations:** 1 National Institute of Materials Physics, 405A Atomistilor Street, 077125 Magurele, Romania; 2 Faculty of Physics, University of Bucharest, 405 Atomistilor Street, 077125 Magurele, Romania; 3 National Institute for Lasers, Plasma and Radiation Physics, 409 Atomistilor Street, 077125 Magurele, Romania; 4 EVGroup, DI Erich Thallner Strasse 1, 4782 St. Florian am Inn, Austria

**Keywords:** nanoimprint lithography, organic photovoltaics, matrix-assisted pulsed laser evaporation, 1,8-diiodooctane, micro-patterning

## Abstract

Lately, there is a growing interest in organic photovoltaic (OPV) cells due to the organic materials’ properties and compatibility with various types of substrates. However, their efficiencies are low relative to the silicon ones; therefore, other ways (i.e., electrode micron/nanostructuring, synthesis of new organic materials, use of additives) to improve their performances are still being sought. In this context, we studied the behavior of the common organic bulk heterojunction (P3HT:PC70BM) deposited by matrix-assisted pulsed laser evaporation (MAPLE) with/without 0.3% of 1,8-diiodooctane (DIO) additive on flat and micro-patterned ITO substrates. The obtained results showed that in the MAPLE process, a small quantity of additive can modify the morphology of the organic films and decrease their roughness. Besides the use of the additive, the micro-patterning of the electrode leads to a greater increase in the absorption of the studied photovoltaic structures. The inferred values of the filling factors for the measured cells in ambient conditions range from 19% for the photovoltaic structures with no additive and without substrate patterning to 27% for the counterpart structures with patterning and a small quantity of additive.

## 1. Introduction

Lately, there is an increasing demand to develop systems that are able to efficiently collect renewable energy. From the available green energy sources, solar is the most promising, being practically inexhaustible [1,2]. Although silicon-based solar cells dominate the photovoltaics market, many studies have focused on fabricating solar cells based on III–V compounds, perovskites, chalcogenide thin films, dye-sensitized, and organic films [3,4,5,6,7,8,9,10,11,12]. Hence, great interest has been given to solar cells based on organic films (small molecules, polymers) due to such features as the diversity of compounds, absorption properties of the organic semiconductors, the requirement of a small quantity of organic material to obtain a thin film, and the possibility to build cheap and eco-friendly light-weight organic photovoltaic (OPV) cells [13,14]. Moreover, different types of applications, such as shading elements or heat shielding for windows, power-generating windows in buildings, photovoltaic green houses, or power-generating displays in mobile phones can be targeted considering the design versatility of the OPV cells [15,16,17,18,19,20,21,22].

In order to become competitive with the inorganic solar cells characterized by high efficiencies (as those based on silicon or gallium arsenide), various approaches have been applied to enhance the electrical parameters of the OPV cells [23,24,25,26]. Thus, a large variety of materials and cells architectures have been studied, and efficiencies over 18% were recently reported [27,28]. However, many efforts are still being made in order to identify (i) high-performance photovoltaic organic materials, (ii) favorable solar cell configurations, (iii) adequate thin film deposition methods, and (iv) patterning designs for electrodes or active layers to improve light harvesting, exciton dissociation, film morphology, and the transport and collection of the charge carriers [29,30,31,32,33,34,35]. In principle, efficient devices can be obtained by optimizing more than one single aspect of the organic solar cell [36].

By definition, an organic photovoltaic cell can be single-layer (one layer of organic compound), dual-layer (two layers: donor and acceptor organic materials as the active layer), or bulk heterojunction (BHJ, dispersed heterojunction blend). It is supposed that the BHJ organic cells combine the advantages of both single- and dual-layer OPV cells by mixing small regions of electron donors and electron acceptors, increasing the interfacial area between them and thus shortening the diffusion length of the exciton. Considered the best way to increase the efficiency of photovoltaic cells, BHJ faces certain problems, such as structural traps, isolated domain formations, and natural demixing due to the fact that it is usually fabricated by blending two components, with different types of molecules in terms of dimension and surface energy, in the same solvent. Usually, the solution is cast onto the transparent electrode by spin coating, which determines an inefficient transport of the charge carriers [37]. Moreover, because of the material loss in both (i) solution preparation (10 to 20 mg/mL of organic material is necessary) and (ii) deposition (by spinning, a significant part of the prepared solution is ejected from the substrate by centrifugal forces), and also due to the limitations of the solvent (low-evaporation-rate solvents cannot be used), different deposition methods have been proposed, such as doctor Blade printing, spray coating, push coating, slot-die, or laser-based techniques like matrix-assisted pulsed laser evaporation (MAPLE) [38,39,40,41,42,43]. Between them, MAPLE does not require highly concentrated solutions (3 mg/mL) and can be used also for water or low-evaporation-rate-solvent-based solutions [44,45].

In order to improve the morphology after the deposition process, various methods are applied, such as thermal treatment or solvent vapor annealing [46,47]. Recently, there is a particular interest in the use of common solvents as additives in the mixture, with the aim of modifying the morphology of the thin film during the deposition step [48]. This small volume of solvent (less than 10%), such as 1,8-diiodooctane (DIO), 1,8-octanedithiol, chloronapthalene, diphenyl ether, or 1-phenylnaphthalene, added to the blend-casting solution is chosen in order to have a large influence on the drying and film formation process to obtain a well-controlled morphology [47,49,50,51]. Usually, they are added in the blends in order to achieve an almost ideal phase separation in the bulk heterojunctions [52,53]. Between them, DIO can increase the carrier’s generation rate, reducing simultaneously the geminate and biomolecular recombination and in this way, enhancing electron mobility [53,54]. Furthermore, in poly(3-hexylthiophene-2,5-diyl (P3HT) and fullerene mixtures, this additive acts as a swelling agent for the P3HT, delaying film solidification due to its low vapor pressure, thereby allowing to the fullerene to enter between the created spaces [52].

An enhancement in light absorption can be achieved by increasing the active layer thickness. However, due to the low mobility of the organic compounds, there are losses associated to the charge recombination, which further decrease the efficiency of the cell structures [55]. Strategies to trap the light and improve the photon absorption involve the use of metallic nanoparticles (NPs), periodically nanostructured electrodes (metallic or transparent), active layers, or antireflection coatings [56,57,58,59]. Between the techniques that can be used for patterning, UV nanoimprint lithography (UV-NIL) is a straightforward texturing technique which can be applied on different rigid or flexible substrates, resulting in a wide range of pattern shapes and sizes over a large area [60,61,62,63].

The purpose of the paper is to investigate, for the first time to our knowledge, the effect of the micro-patterned (pyramidal shape) transparent electrode and DIO additive on the properties of photovoltaic structures based on P3HT: [6,6]-Phenyl-C71-butyric acid methyl ester (PC70BM) thin films deposited by MAPLE.

The choice of the organic materials was based on the fact that, from the available polymers, P3HT and PC70BM (or PC71BM) are the most studied [9,52,56,64,65]. The donor P3HT is characterized by a high mobility (0.1 cm^2^ V^−1^ s^−1^) and a reduced band gap (1.9 eV) [55,66]. On the other hand, fullerene and fullerene derivatives have been frequently used as acceptors due to their mobility, specifically PC60BM, which was used frequently in combination with P3HT [64,67,68,69]. However, as a consequence of its poor absorbance in the visible part of the solar spectrum, PC60BM was replaced with PC70BM [18,39,55,67,70,71,72]. Additionally, toluene was used as solvent in order to compare the results with the only ones reported in the literature referring to both P3HT and fullerene coatings for photovoltaics solar cells using MAPLE [43]. Furthermore, toluene is a non-halogenated solvent, like xylene or 1,2,4-trimethylbenzene, which in combination with 0.5% DIO additive, gives the best performances, similar to those obtained for chloroform, a solvent from the halogenated family [73].

## 2. Materials and Methods

The organic materials, the regioregular P3HT donor (RR 97.3%-M1010), and the fullerene electron acceptor PC70BM (purity 99%-M114) were acquired from Ossila Ltd. (Sheffield, UK). The 1,8-diiodooctane (DIO) additive, with 98% purity and copper stabilizer, and the toluene solvent were purchased from Sigma-Aldrich (St. Louis, MO, USA). The primer and photoresist are provided by EVGroup (St. Florian, Austria). All chemicals were used without further purification.

### 2.1. Preparation of the Photovoltaic Cell Structures on Flat and Micro-Patterned Glass Substrates

**Figure 1:**
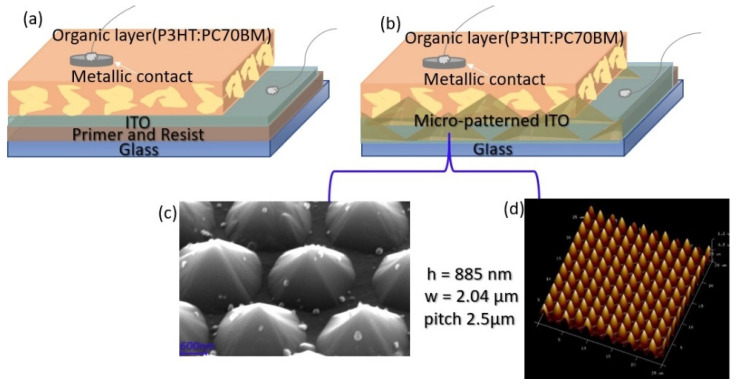
Schematic representation of the photovoltaic cell structures developed on (**a**) flat and (**b**) micro-patterned ITO electrodes, with (**c**) SEM and (**d**) AFM micrograph on 25×25 μm area, showing details of the imprinted pyramids characteristics.

A schematic representation of the photovoltaic cell structues fabricated on flat and micro-patterned glass substrates is shown in Figure 1. The processes involved in their fabrication are described below.

#### 2.1.1. Micro-Patterning Substrates by UV-NIL

The glass micro-patterned substrates were fabricated by the UV-NIL technique. The detailed process from master until stamp fabrication was described in our previous paper focused on the transfer of the patterns on silicon p-type substrates [74]. In the present study, the same working stamp was used in order to fabricate pyramids on glass substrates. Hence, the glass substrates were cleaned and coated with the primer EVG PrimKRD prior to the deposition of the resist. The primer, consisting of a sub-10 nm layer, ensures a strong adhesion in order to avoid the delamination of the imprint when the stamp is removed. An EVG UV-A2, 300 nm resist was used as negative photoresist, a layer thickness of 276 nm being deposited at 2000 rpm for 60 s. The residual layer was ~20 nm in thickness after the imprint process. The nanoimprint process was performed with an EVG^®^ 7200 automated UV Nanoimprint System using for resist curing a 365 nm wavelength LED lamp at 300 mW/cm^2^ for 60 s.

The final shape and characteristics of the pyramids imprinted onto the resist are presented in Figure 1c,d. The pyramids have a base width (w) of 2.04 μm and a height (h) of 885 nm, the distance between the centers of the two neighboring pyramids being 2.5 μm.

#### 2.1.2. Transparent Electrode Deposition by Pulsed Laser Deposition (PLD)

The indium thin oxide (ITO) transparent conductive electrode was deposited on flat and micro-patterned glass substrates by the Pulsed Laser Deposition (PLD) technique using a KrF* excimer laser source (ComplexPro 205, λ = 248 nm, τ_FWHM_ = 25 ns from Coherent Inc., Santa Clara, CA, USA) and an ITO (In_2_O_3_:SnO_2_ = 90%:10% wt.) solid target (SCI Engineered Materials Inc., Columbus, OH, USA).

The experimental parameters, 1.2 J/cm^2^ laser fluence, 7000 laser pulses, 1.5 Pa oxygen 6.0 pressure inside the deposition chamber, and a 5 cm distance between the target and the substrate, were chosen according to our previous studies in order to obtain a working thickness of the ITO layers of about 340 nm [75,76,77].

#### 2.1.3. Active Layer Deposition by MAPLE

The active layer was deposited by MAPLE, a laser-based deposition technique that uses a frozen target made of organic compound dissolved in a solvent, which can absorb the radiation of the laser involved in the organic layer deposition. In our case, the frozen target was made from P3HT:PC70BM (at different concentrations) dissolved in toluene or in a toluene: DIO mixture, as mentioned in Table 1. Reference solutions containing only P3HT or PC70BM were also prepared. The DIO quantity was chosen based on the study focused on the slot-die deposition [42], which emphasized that the charge transport to the electrodes is hindered at concentrations higher than 0.5% DIO, the best improvements being achieved at 0.25% DIO concentration. In the present study, the choice was also justified by the fact that at higher DIO concentrations, some traces of additive can remain laser-transferred in the active layer due to its high boiling point (332.5 °C) and low vapor pressure (0.375 $\times 10^{-3}$ mbar). These traces can affect the OPV cell stability [37].

For all the targets used in the MAPLE deposition, the total solution (blend) composition was kept at 3 mg/mL. The blend compositions were chosen in such a manner that the amount of P3HT reaches the threshold of its solubility in toluene, i.e., 1.4 mg/mL in the case of P3HT: PC70BM at a 1:0.7 ratio.

The organic layers were deposited on the substrates placed at a 5 cm distance from the target by irradiating the frozen solution with a KrF* excimer laser (the same as for the PLD) at 5 × 10^−4^ mbar pressure inside the chamber and room temperature. The laser energy distribution in the beam cross-section was improved by means of a laser beam homogenizer. As a result, a rectangular spot with an area of 26 mm^2^ and pulse energy of 65 mJ assured a laser fluence of 250 mJ/cm^2^. For all MAPLE depositions, the coatings were deposited by applying 50,000 pulses at a laser frequency of 20 Hz.

In the same MAPLE deposition cycle, besides ITO substrates, the organic layers were also deposited on silicon and glass substrates for different investigations. Furthermore, from each solution involved in the preparation of the frozen targets, organic layers were deposited on silicon substrates by the drop-cast method in order to be used as reference in the FTIR measurements. The thickness of the films was obtained as the average value of scans in four different points by using an Ambios Technology XP 100 profilometer (Ambios Technology Inc., Santa Cruz, CA, USA).

#### 2.1.4. Top Metallic Electrode Deposition by Vacuum Thermal Evaporation (VTE)

The photovoltaic cell structures were finalized by depositing a top metallic electrode consisting of a thin layer of 1.5 nm lithium fluoride (LiF) and a layer of 100 nm aluminum (Al) by VTE using a Tecuum AG, VCM600-V3-80 (Winterthur, Switzerland) at a residual pressure of 1.6 × 10^−6^ mbar using a shadow mask with holes having 28 mm^2^.

The samples investigated in the present study were labeled depending on the composition of the solution, the deposition technique, and the type of the substrate, as specified in Table 2. Furthermore, for comparison reasons, the references consisting of P3HT and PC70BM films deposited on silicon and glass substrates were assessed.

### 2.2. Characterization Techniques

#### 2.2.1. Fourier Transform Infrared Spectroscopy (FTIR)

Each organic material has a vibrational “fingerprint” that can be highlighted through FTIR measurements. Thus, in order to evaluate if the chemical structure of the organic compounds was preserved during the MAPLE deposition, a set of measurements on silicon substrates was performed using an IRTracer-100 (Shimadzu, Kyoto, Japan) spectrometer, working in transmission mode, in the range 450–3100 cm^−1^ with 4 cm^−1^ resolution.

#### 2.2.2. Atomic Force Microscopy (AFM) and Field Emission Scanning Electron Microscopy (SEM)

The roughness of the deposited organic thin films was evaluated by AFM using a MultiView 4000 system (Nanonics Imaging Ltd., Jerusalem, Israel) with a scan area of 40 × 40 μm.

The morphology of the organic layers deposited on flat and patterned substrates was evaluated using an ApreoS instrument (Thermo Fisher Scientific, Waltham, MA, USA), working in high vacuum and with an acceleration voltage of 10 kV.

#### 2.2.3. Spectroscopic Ellipsometry Measurements

Specular reflectance measurements at different incidence angles were performed using a variable angle spectroscopic ellipsometer (Woollam V-VASE). The reflectance data were acquired in the 500–1000 nm range with 0.1 step, at two different angles: 35 and 45°, having the polarizer angle set at 45°.

#### 2.2.4. UV-VIS and Photoluminescence (PL) Spectroscopy Measurements

The optical properties of the organic layers were investigated by UV–VIS spectroscopy using an Evolution 220 UV-VIS spectrophotometer equipped with Insight software (Thermo Scientific, Schwerte, Germany). The UV-VIS measurements were performed in absorption and transmission modes, in the 190–1100 nm range with 0.2 s integration time and with a scan speed of 300 nm/min.

The PL measurements were performed using an Edinburgh Instruments spectrometer with a 450 W Xe lamp. The PL spectra were recorded in the 450–850 nm range, at 435 nm excitation wavelength.

#### 2.2.5. Current Density-Voltage (J-V) and External Quantum Efficiency (EQE) Measurements

The electrical characteristics (J-V) and (EQE) of the organic heterostructures were acquired in dark and solar simulation conditions (AM 1.5, incident power density 100 mW/cm^2^) using a home-made set-up composed of a Keithley SourceMeter 2400 model, a Newport Oriel monochromator, and a Newport Oriel solar simulator controlled by a computer through a LabVIEW interface.

## 3. Results and Discussion

**Figure 2:**
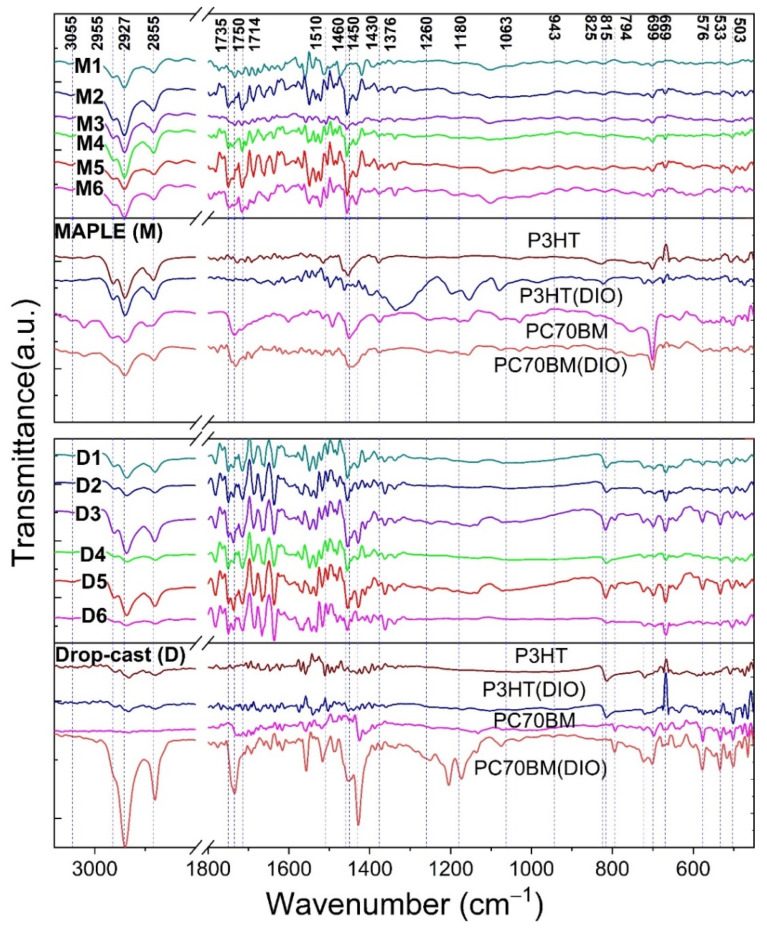
FTIR spectra of the drop-cast (D) and MAPLE (M) films onto silicon substrates. The index indicates the solutions (according to Table 1) used as target in their deposition.

The preservation of the chemical structure of the organic compounds used in the MAPLE deposition of P3HT:PC70BM thin films was evaluated by FTIR analysis. Thus, in Figure 2 are presented FTIR spectra of the MAPLE organic thin films as a mixture and as single component versus drop-cast organic layers deposited on silicon substrates.

In the 2960–2700 cm^−1^ region, the absorptions are usually assigned to C-H stretching vibration from methylene and methyl of the alkyl chains from the thiophene rings of P3HT [77,78]. Thus, the drop-cast and MAPLE mixed layers present the antisymmetric CH_3_ stretching mode at around 2955 cm^−1^ and the antisymmetric CH_2_ stretching around 2927 cm^−1^, similar to the ones noted in the FTIR spectra of the P3HT and PC70BM reference thin films. The C-H stretching vibration corresponding to the symmetric CH_2_ stretching mode is observed at around 2855 cm^−1^. The very weak band at around 3055 cm^−1^, barely noted in the MAPLE films but broader in the drop-cast ones, corresponds to =CH stretching vibration [79].

The intensity of the band located at 1735 cm^−1^ associated to the C=O stretch of the ester bond of PC70BM is decreased in comparison to the FTIR spectra of the pristine fullerene reported in the literature as a consequence of the formation of a hydrogen bond. Furthermore, the absorption bands from 1750 and 1714 cm^−1^ can be related to the formation of the photodegradation products in the air, as was reported for the spin-coated layers [80,81]. The characteristic peaks of PC70BM can be observed at 1430 cm^−1^ (CH_2_ bending vibration) and 1180 cm^−1^ (O-C single bond vibration), the former missing completely in our “blend coatings” deposited by drop-cast and MAPLE on silicon substrates [71,82]. In the case of the absorption bands of P3HT, the principal ones correspond to -CH_3_ vibration of the end groups noted at 1376 cm^−1^, to C=C thiophene at 1460 cm^−1^, and to antisymmetric C=C stretching of the backbone at 1510 cm^−1^ [78,82]. The presence of the small absorption band near 1260 cm^−1^ assigned to the epoxy group (C-O-C) in the reference drop-cast films justifies the degradation of the film due to the residual additive. The absence of this absorption band for the FTIR spectra of the MAPLE-deposited films demonstrates that the additive molecules are not trapped inside during the MAPLE process [37].

In the 1000–400 cm^−1^ region, the characteristic absorption bands linked to the functional groups of both organic compounds can be identified. In the case of P3HT, the absorption band associated to C-H out-of-plane deformation vibration is observed at 815 cm^−1^ for all drop-cast samples and at 825 cm^−1^ for the MAPLE samples, broader in the case of MAPLE films in comparison to the drop-cast layers [83,84,85]. The absorption band is more intense for the drop-cast films deposited from the solutions without DIO, the miscibility of the two organic compounds being lower than that obtained in the solutions containing DIO as additive. Moreover, at 722 cm^−1^ can be observed the band associated to the CH_3_ rocking vibration [85] or to the characteristic absorption of the sulfur atom on the polythiophene ring [81], and at 533 cm^−1^ the band due to the vibrations in the fullerene cage [86].

The topography and the morphology were analyzed for the samples that presented a similar thickness of the organic layer (I3, I3nano, I2, and I2nano samples) by AFM and SEM. The thickness of the organic layers deposited onto ITO and ITO micro-patterned electrodes is shown in Table 3. The thickness of the organic layers deposited by MAPLE on glass and silicon substrates is similar to the thickness of the corresponding organic layer deposited onto the ITO flat electrode (I1–I6) of the same target solution, since they were deposited in the same deposition process.

**Figure 3:**
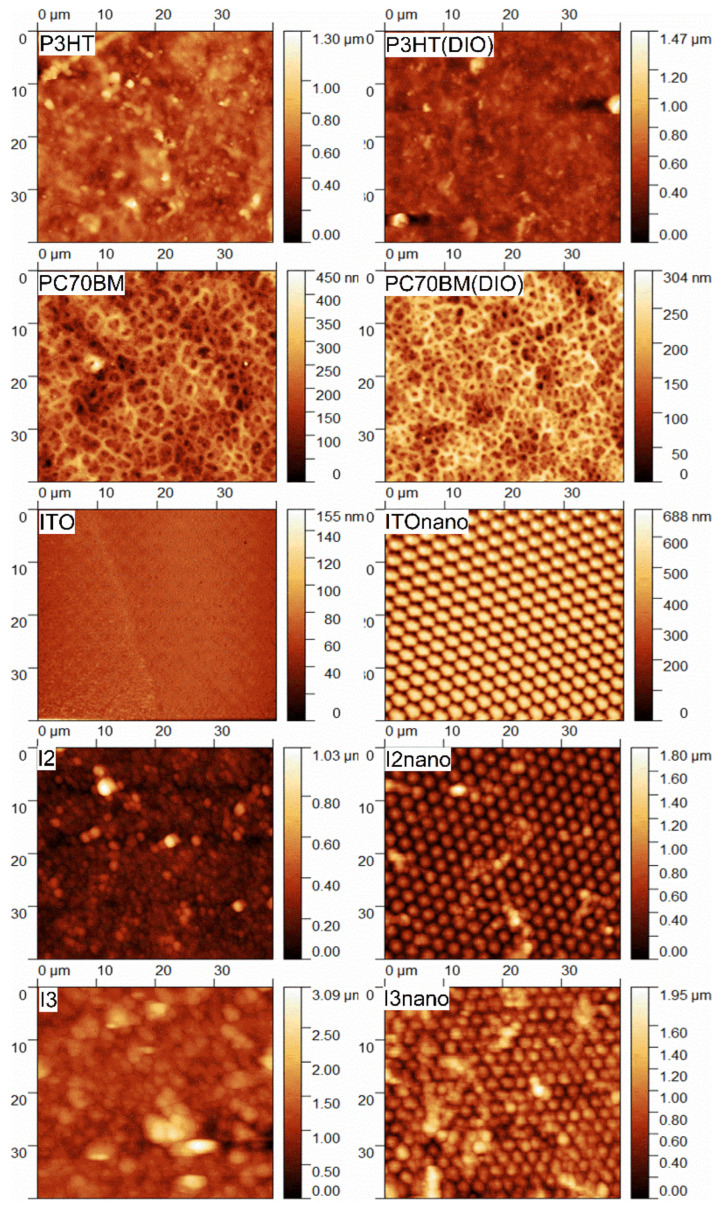
AFM images of P3HT and PC70BM as reference films deposited by MAPLE, flat ITO, and patterned ITOnano substrates and MAPLE blend layers: I2, I2nano deposited from P3HT:PC70BM (1:0.7) and I3, I3nano deposited from P3HT:PC70BM (1:1).

The AFM images (Figure 3) of the P3HT films prepared from the targets with/without additive show that the molecular aggregation effect in the films is lower when a quantity of 0.3% DIO is added. 

This result is in conformity with the values obtained for the root mean square roughness (RMS) and roughness average (Ra) parameters, which decrease from 112 nm to 105 nm and from 84 nm to 70 nm, respectively, as can be seen in Table 4. The additive also prevents the P3HT from drying faster than the fullerene during the deposition process, acting as a swelling agent and therefore facilitating the charge separation [52,87]. The PC70BM films deposited by MAPLE from toluene solution have a spongious topography that is also preserved when the additive is added in the solution. The only difference is the fact that PC70BM(DIO) films present a more packed morphology, reflected also by the reduction in the RMS value from 49 nm to 44 nm and of the Ra value form 39 nm to 36, as reported in Table 4.

The active layer deposited on flat substrate (I3 sample (P3HT:PC70BM (1:1)) is characterized by a rugosity given by the presence of large grains, a similar result being reported for PC70BM deposited by vacuum evaporation [72]. For the I2 sample (P3HT:PC70BM (0.7:1) with DIO), the AFM image (Figure 3) confirms that the addition of the DIO additive in the blends suppresses the formation of large fullerene domains, which are critical for harvesting excitons. Thus, the use of a small concentration of additive improves the RMS and Ra parameters of the organic thin films deposited by the MAPLE technique (Table 4).

**Figure 4:**
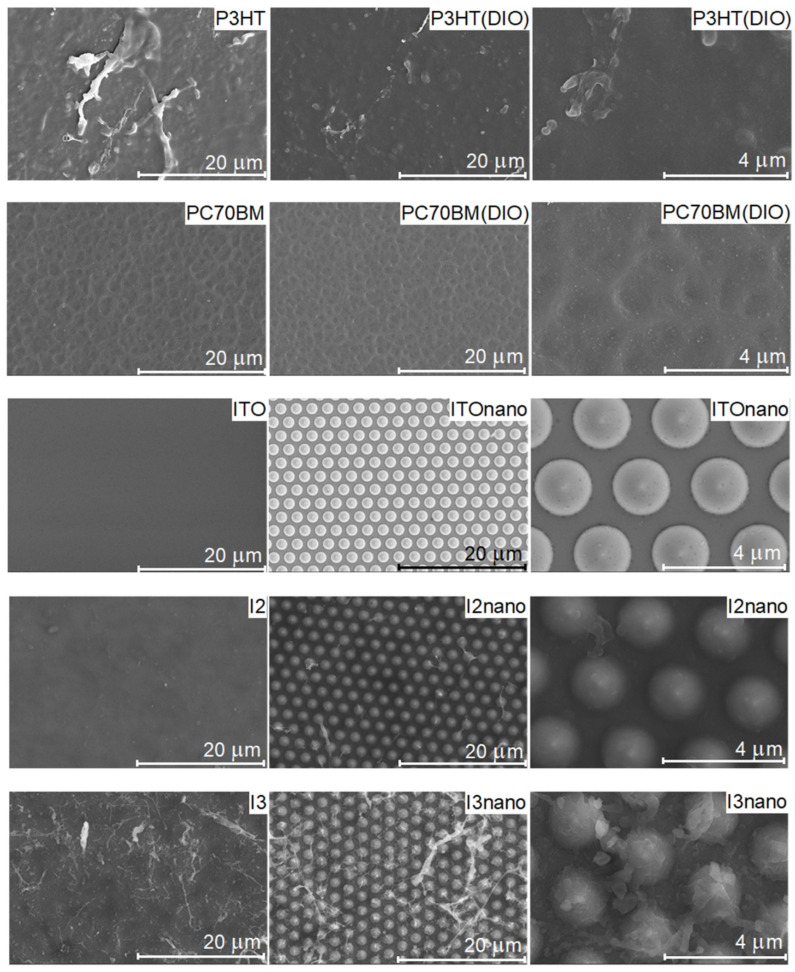
SEM images (at two magnifications) of the P3HT and PC70BM reference films deposited by MAPLE, flat ITO, and patterned ITOnano substrates and MAPLE blend layers: I2, I2nano deposited from the target P3HT:PC70BM (1:0.7) and I3, I3nano deposited from the target P3HT:PC70BM (1:1).

The SEM images (Figure 4) evidenced that the samples prepared from targets without DIO (I3, I3nano samples) present fibrillar nanocrystals of P3HT similar to the ones noted in the case of P3HT film. Less widespread nanocrystals have also been observed in the samples with DIO (I2, I2nano samples). Furthermore, the SEM images of the mixed films deposited from the target with DIO additive reveal a combination of the morphology of both organic compounds, suggesting that the PC70BM is intercalated within P3HT. As for the films without DIO, the P3HT fibrils are visible on the surface. In the case of the films deposited onto the micro-patterned substrate, the pattern’s topography is maintained.

Hence, the SEM images and AFM analysis prove that the use of DIO as an additive shrinks the fullerene dispersion without altering composition.

**Figure 5:**
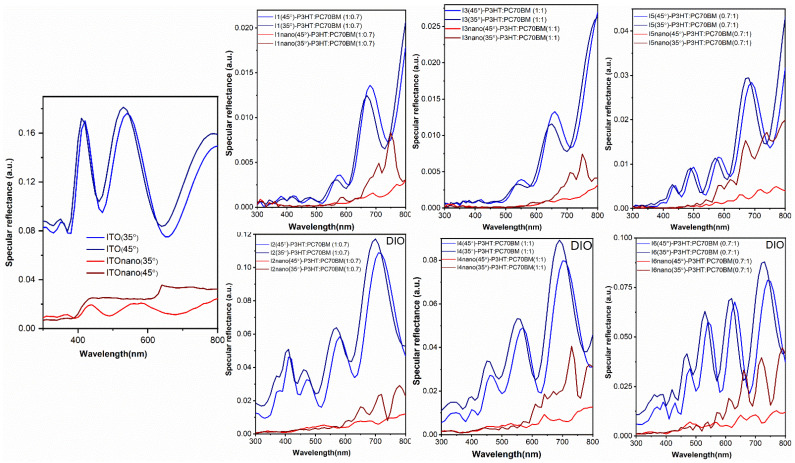
Specular reflectance curves (at two different incidence angles) of the flat and patterned ITO electrodes and of the organic films deposited by MAPLE on these substrates from the targets prepared without and with DIO.

In order to see the influence of the micro-patterns’ specular reflectance, measurements were carried out at two different angles on the flat and patterned electrodes and on the organic heterostructures fabricated on them (Figure 5).

From the specular reflectance curves of the flat and patterned ITO electrode can be seen that the micro-patterns induce lower specular reflectance compared to the flat surface electrode. The shape of the specular curves of the ITO flat electrode is almost independent for the two incident angles. Furthermore, the interference fringes of the flat electrode are given by the thickness of the ITO deposited onto the glass substrate. Interference fringes are also visible in the case of the micro-patterned ITO electrode for the specular reflectance curve obtained at 35°, but they are missing or distorted in the specular reflectance curve recorded at 45°. Therefore, the specular reflectance of the micro-patterned structures is dependent of the incidence angle of the radiation. The reduction in the specular reflectance in the case of the ITO micro-patterned electrode is due to the fact that the incident light is reflected not only from the top and bottom surface of the pyramids, but also by the lateral walls. Therefore, additional reflections and scattering are taking place in the case of the micro-patterned electrode, which induce reflection reduction and increased absorbance.

The discussion regarding the reduction in the specular reflectance due to micro-patterns is also valid for all the analyzed organic films. Furthermore, the organic films deposited by targets containing DIO (I2, I4, I6 samples) are characterized by higher reflectance compared to the organic films deposited from frozen solution without DIO (I1, I3, I5 samples). This can be linked to the increased thickness and rugosity of the samples deposited from the solutions without additive, as was confirmed by the AFM analysis (Table 4). Higher values of these parameters are due to the molecular aggregation of the P3HT polymer and to the large fullerene domains, as was confirmed by SEM analysis (Figure 4). Regarding the P3HT concentration, it can be noted that higher concentration lowers the film reflectance. The result can be explained by the tendency of the P3HT to create films with higher rugosity when deposited by MAPLE from targets with toluene as solvent, compared to the PC70BM (see Table 4).

**Figure 6:**
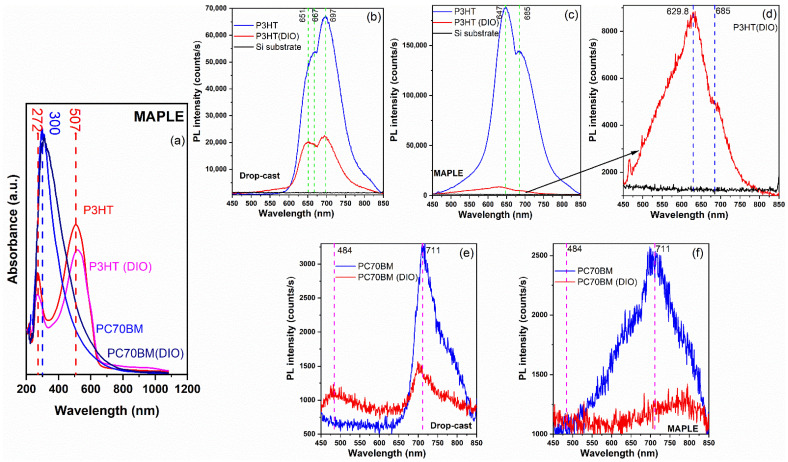
(**a**) UV-VIS and (**b**–**f**) PL spectra of the P3HT and PC70BM thin films deposited by MAPLE (**a**,**c**,**d**,**f**) and drop-cast (**b**,**e**) with and without DIO on glass and silicon substrates, respectively.

**Figure 7:**
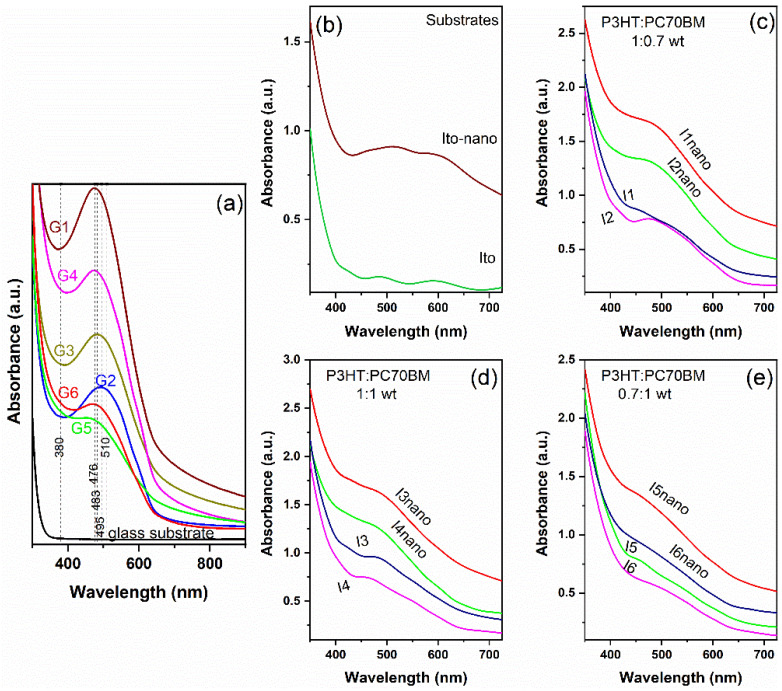
UV-VIS spectra of: (**a**) the thin films deposited on glass substrate by MAPLE (G1–G6), (**b**) flat and patterned ITO substrates and (**c**–**e**) thin films deposited on flat and patterned ITO substrates by MAPLE from targets with different P3HT:PC70BM weight ratio; the index indicates the samples (according to Table 2).

The optical properties of each organic component were analyzed in order to evaluate their contribution to the optical features of the mixed layers (Figure 6 and Figure 7). The absorbance spectra of the pristine P3HT and PC70BM layers deposited by MAPLE (with and without DIO) are given in Figure 6a. Usually, P3HT features a large absorption band with three maxima at 510, 550, and 600 nm [54,57]. However, the P3HT films deposited by MAPLE are characterized in the visible domain by a wide band between 330 to 650 nm. The addition of 0.3% DIO additive decreases the P3HT films’ absorbance. The fullerene film is characterized by a stronger absorbance between 300 to 600 nm, with the maximum in the UV light range (300 nm), owing to the highest occupied molecular orbital (HOMO)–lowest unoccupied molecular orbital (LUMO) transitions [88]. Therefore, it is important not to neglect the photocurrent generated by the light absorbed by the PC70BM in the discussion regarding the performances of the photovoltaic structures. In the case of PC70BM, the addition of the DIO additive does not influence the absorbance spectra; the only change observed is a slight narrowing of the domain.

The absorption spectra of P3HT:PC70BM films with different weight ratios deposited by MAPLE on glass substrates (G1:G6) are shown in Figure 7a. Hence, the absorption range of the deposited films is situated between the absorbance ranges of the two organic components, at around 500 nm, near the maxima attributed to the π-π^*^ transition. The absorbance spectra of the P3HT:PC70BM films maintain the shape of the absorbance spectra of the P3HT film. The influence of the fullerene addition is highlighted by the tendency to increase the absorbance towards smaller wavelengths. The absence of the 550 and 600 nm shoulders characteristic of P3HT in the pristine and mixed films deposited by MAPLE can imply the alteration of the degree of the interchain order [89].

In the case of samples with the quantity of P3HT equal to or lower than that of fullerene, prepared using DIO as additive (G4 and G6), the absorption is higher than that of the samples prepared without DIO (G3, G5). This can be explained by the fact that the PC70BM contribution, which is not strongly affected by the additive addition in its absorbance, is higher compared to the films with a higher concentration of P3HT (G1, G2) due to the π-π^*^ interactions [90]. Furthermore, when the quantity of P3HT exceeds the quantity of PC70BM, the absorbance of the films without DIO is higher than the absorbance of the films with DIO. This result reinforces the statement made after the FTIR analysis that the addition of DIO as additive increases the solubility of fullerene and decreases the absorbance of the P3HT films.

Considering that the MAPLE organic layers will be integrated in structures with patterned electrodes in order to increase the efficiency of the photovoltaic heterostructures, the UV-VIS spectra of flat and patterned ITO substrates were analyzed (Figure 5). As expected, the spectra highlighted the fact that the absorption is higher in the case of the patterned substrate, due to the multiple reflections on the pyramid patterns walls, in agreement to the specular reflectance data (Figure 5). The same tendency remains for all heterostructures deposited on the micro-patterned ITO substrates. All the UV-VIS spectra preserve the shape of the used substrate and the visible absorption features of the P3HT:PC70BM blends, meaning a large absorption band between 400–600 nm as a sum of the individual P3HT and PC70BM absorptions reported in [43]. In terms of the fullerene composition, it can be observed that on the ITO substrates the 1:1 ratio mixed film (I3, I3nano, I4, I4nano samples) presents the maximum absorbance. In accordance with the FTIR data (Figure 2), the results confirm that no damaging phenomena concerning the chemical structures of the organic compounds occur during their MAPLE deposition [83].

In order to analyze the influence of both the deposition method and the additive on the emission properties of the organic blends, firstly the PL spectra of the pristine P3HT and PC70BM films deposited by MAPLE from targets with and without DIO were analyzed in comparison with those obtained by drop-cast (Figure 6b–f).

The P3HT polymer film presents a strong red emission peaking at 667 and 697 nm when it is deposited by drop-cast (without DIO additive). The addition of DIO results in a strong quenching of the P3HT film emission and a slight shift of the position of the peak from 667 nm to 651 nm (Figure 6b). It can be noted that the emission of the P3HT film deposited by MAPLE is higher than that of the film deposited by drop-cast. Moreover, the peaks are shifted to lower wavelengths at 647 and 685 nm (Figure 6c–d). The shape of P3HT film deposited by MAPLE mirrors the shape of the drop-cast film. The quenching observed due to the DIO addition is stronger in the case of the MAPLE deposition.

The pristine PC70BM films deposited by both drop-cast and MAPLE technique (Figure 6e,f) are characterized by a low intensity emission band centred at 711 nm, because of the low quantum yields of the almost spherical fullerene molecules [91]. The emission intensity seems to decrease slightly for the films deposited by MAPLE (Figure 6f). Furthermore, the quenching resulting by the addition of the DIO additive is not as strong as in the case of P3HT films.

The PL spectra of the mixed films with and without DIO deposited by drop-cast on silicon substrates (D1–D6) show red emission with peaks at 640 and 697 nm, attributed to P3HT [83]. Compared to the pristine P3HT film, the maximum from 667 nm is shifted by 27 nm to lower wavelengths. Moreover, the photoluminescence emission intensity is slightly higher than that of the pristine PC70BM film deposited by drop-cast and significantly weaker than that of the pristine P3HT film. This is a consequence of the fast dissociation of excitons to free charges at the interfaces of the donor and acceptor domains [92]. Since the PC70BM has the emission peak at around 711 nm, the 697 nm emission peak from the drop-cast samples can be a combination of the maxima of both P3HT and PC70BM compounds [45,93].

In the PL spectra of the films deposited by MAPLE on silicon substrates (M1–M6), it can be observed that the emission bands become larger; the dominant emission peaks are still visible, but slightly shifted for the samples obtained using an equal or higher quantity of P3HT without DIO additive (M1, M3). This can be explained by the fact that usually the emission is strongly quenched by the addition of fullerene [70]. Moreover, the structures prepared using DIO reveal higher PL quenching compared to the heterostructures without DIO, implying usually a better charge transfer, as can be noted in I2, I2nano; I4, I4nano; and I6, I6nano samples [53].

**Figure 8:**
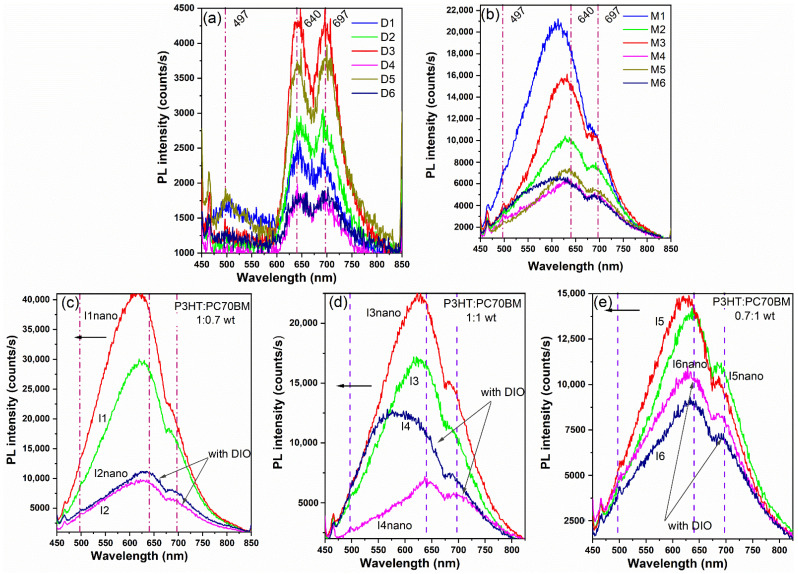
PL spectra of the layers deposited on (**a**) silicon substrates by drop-cast (D1–D6) and (**b**) MAPLE (M1–M6) and (**c**–**e**) thin films deposited on flat and patterned ITO substrates by MAPLE from targets with different P3HT:PC70BM weight ratio; the index indicates the samples (according to Table 2).

The PL spectra of the sample deposited onto the flat and patterned ITO electrodes presented in Figure 8c–e reveal similar emission features to those of the layers deposited by MAPLE on silicon substrates. Furthermore, the emission intensity of the films is proportional with the quantity of the P3HT, meaning a higher PL signal at higher P3HT concentration. Moreover, it can be observed that the patterns slightly increase the PL intensity for the sample with a higher concentration of P3HT.

The current–voltage characteristics (I–V) in the dark of the evaluated samples were drawn at room temperature, using the same experimental set-up as for the standard AM 1.5 condition measurements. It can be noted the non-linear behavior of all structures, which is specific to the diodes with not very high rectification factors and relative asymmetry.

**Figure 9:**
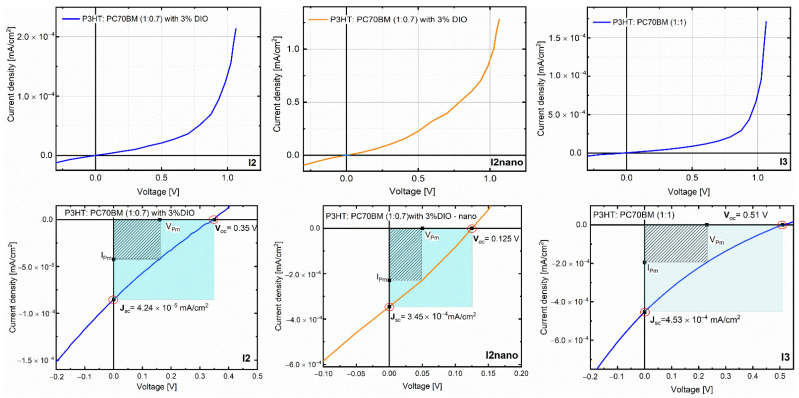
J-V characteristics of the structures based on MAPLE-deposited organic layers on ITO and ITO micro-patterned electrodes under dark conditions and solar illumination.

The photo-electrical response of the prepared samples was analyzed by current–voltage curves in the solar simulator conditions, AM 1.5, P_in_ = 100 mW/cm^2^ (Figure 9, bottom part). One may see that the photo-generated current density in short-circuit for the sample with a larger quantity of P3HT and DIO deposited on micro-patterned ITO substrates has similar values as for the conventional devices based on P3HT and PC70BM, 1:1 wt.%, and improved performance compared to similar devices obtained on flat substrates. Considering that the optical results showed an improved absorption for the samples without DIO when the P3HT quantity was larger than the PC70BM one, a possible explanation could be related to a better charge collection to electrodes. It can be assumed that for the micro-patterned ITO substrates, which have a highly arranged morphology, the transportation paths of photo-generated charge carriers are well defined, so the leakage ways are reduced. On the other hand, the open-circuit voltage V_oc_ demonstrates the opposite behavior; it has higher values for the structures deposited on flat substrates. The open-circuit voltage strongly depends on the saturation current, which is a measure of the recombination. Thus, most probably the cause of the decrease in V_oc_ is the high recombination due to an increased number of interfaces for the micro-patterned devices. This assumption is supported by the action spectra, which are the dependence of the external quantum efficiency (EQE) on the incident wavelength. Furthermore, it was demonstrated that by adding DIO, the structure usually exhibits lower open-circuit voltages, but higher photocurrents and fill factors compared to the cells without additive or thermal treatment [68]. In our case, the values of the fill factors (FF) for the structures are FF_I2_ = 23%, FF_I2nano_ = 27%, and FF_I3_ = 19%, in agreement with the results published in [43,68,89]. The differences are not so notable in the case of the lower quantity of the DIO additive, but it is expected that the improvements will be more visible by increasing the additive concentration [68]. As a general observation, the J-V characteristics under illumination show a very low photovoltaic effect, whereas DIO additive and the use of a micro-patterned substrate have a positive influence on the properties of the investigated films.

**Figure 10:**
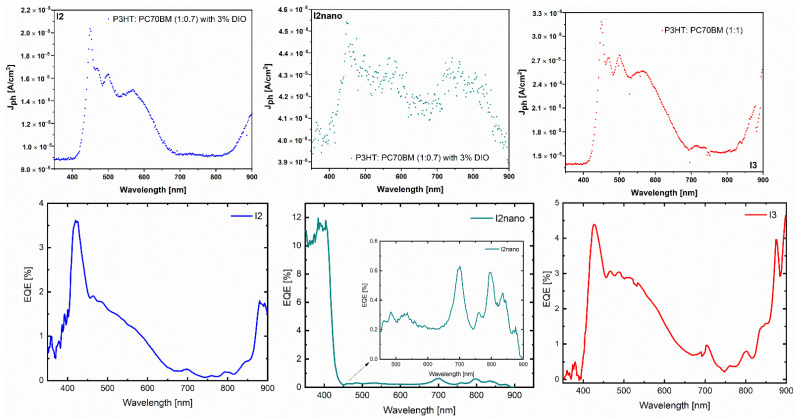
The photocurrent shape of the structures based on MAPLE-deposited organic layers on ITO and ITO micro-patterned electrodes and their external quantum efficiency (EQE) dependence on the incident wavelength (bottom part of the figure).

The general definition of the EQE is the ratio between the number of collected photo-generated charge carriers and the number of incident photons for a given wavelength. Comparing the obtained results for I2, I2nano, and I3 samples (Figure 10, bottom part), it can be clearly noted that large and well-defined maxima in the EQE spectrum are related to good values of V_oc_, i.e., 0.51 V for I3, which reinforce the idea of low recombination at the interface. In order to identify more easily the signature of P3HT, in the manuscript we plot and add generated photocurrent vs. wavelength graphs (Figure 10, upper part). For the conventional P3HT:PC70BM (1:1) sample without DIO, the specific broad absorption range of the active layer between 400–600 nm is observed. The PC70BM maximum at 380 nm and P3HT peaks around 510 nm and 550 nm and the shoulder at 600 nm can also be easily seen. The addition of DIO narrows the specific absorption range by flattening the maxima assigned to P3HT. Again, the observation made asserts the FTIR results.

Comparing the structure I3 with the one reported in [89] fabricated by the spin-coating method and using fluorine-doped tin oxide (FTO) and poly(3,4-ethylenedioxythiophene)-poly(styrenesulfonate) (PEDOT:PSS) as a transparent electrode, the value of the fill factor for the ratio 1:1 P3HT:PCBM is almost similar, 17%, with that obtained in our study, 19%, for the same weight ratio. In terms of the same deposition technique for the organic films, MAPLE, there is a reported value for the FF equal to 28% for the P3HT:PCBM bilayer structure [43], similar to that obtained in our study, 27%, when a small amount of DIO additive was added and a micro-patterned substrate was used as an electrode.

## 4. Conclusions

Thin films based on P3HT:PC70BM with different organic composition (with or without DIO as an additive in the blend solution) were deposited by MAPLE (for the first time, to our knowledge) on flat and pyramidal-patterned substrates in order to evaluate their potential application in OPV cells. The FTIR analysis confirms that the chemical structure of organic compounds is preserved during the overall process (formation of the blend solution, frozen target fabrication, and laser evaporation process) and that the DIO additive molecules are not trapped inside the organic film.

The UV-VIS and PL spectra of all investigated organic films deposited by MAPLE reveal the typical absorption and emission bands of both components (P3HT and PC70BM). Moreover, the PL spectra of films deposited by MAPLE evidenced a change in their shape together with a slightly shifted position of emission maxima in comparison to those of films prepared by drop-cast. Regarding the influence of DIO additive and micro-patterned ITO electrodes on the optical properties of the deposited organic films, the following aspects must be emphasized: (i) the UV-VIS spectra show that both increase the absorption in the visible domain; (ii) the addition of 0.3% DIO results in a strong quenching of the P3HT emission and in a lower influence on PC70BM emission; (iii) the ITO micro-patterning increases the emission of the films with a higher or equal concentration of P3HT; (iv) the specular reflectance spectra reveal that the addition of DIO increases the reflectance of the blend films, while the electrode micro-patterning decreases it.

Although the photovoltaic response was very low, the preliminary J-V characteristics of the organic structures recorded under illumination show that the addition of DIO additive in the blend solution decreases the open-circuit voltage (V_OC_), while the value of the short-circuit current density (J_SC_) is comparable for all the investigated structures. The photovoltaic structures fabricated involving both DIO additive and micro-patterned ITO substrates emphasized the highest filling factor. Therefore, it is expected that by optimizing the MAPLE deposition parameters in order to decrease both the thickness and the rugosity of the active layer, as well as by structure encapsulation, an enhancement of the performances of the OPV cell may be achieved.

## Figures and Tables

**Table 1:** Blend composition used in the fabrication of MAPLE targets for the active layer deposition.

Target	Solution Composition	
	P3HT:PC70BM Ratio	DIO Ratio in Toluene
1	1:0.7 wt.	0%
2	1:0.7 wt.	0.3%
3	1:1 wt.	0%
4	1:1 wt.	0.3%
5	0.7:1 wt.	0%
6	0.7:1 wt.	0.3%

**Table 2:** Labels of the deposited organic layers.

Solution Composition	Deposition Type	Substrate Type	Structure	Sample Label
1	Drop-cast	silicon	P3HT:PC70BM (1:0.7)/Si	D1
	MAPLE	silicon	P3HT:PC70BM (1:0.7)/Si	M1
		glass	P3HT:PC70BM (1:0.7)/glass	G1
		ITO *	P3HT:PC70BM (1:0.7)/ITO	I1
		ITO nano #	P3HT:PC70BM (1:0.7)/Micro-patterned ITO	I1nano
2 (with DIO)	Drop-cast	silicon	P3HT:PC70BM (1:0.7)/Si	D2
	MAPLE	silicon	P3HT:PC70BM (1:0.7)/Si	M2
		glass	P3HT:PC70BM (1:0.7)/glass	G2
		ITO *	P3HT:PC70BM (1:0.7)/ITO	I2
		ITO nano #	P3HT:PC70BM (1:0.7)/Micro-patterned ITO	I2nano
3	Drop-cast	silicon	P3HT:PC70BM (1:1)/Si	D3
	MAPLE	silicon	P3HT:PC70BM (1:1)/Si	M3
		glass	P3HT:PC70BM (1:1)/glass	G3
		ITO *	P3HT:PC70BM (1:1)/ITO	I3
		ITO nano #	P3HT:PC70BM (1:1)/Micro-patterned ITO	I3nano
4 (with DIO)	Drop-cast	silicon	P3HT:PC70BM (1:1)/Si	D4
	MAPLE	silicon	P3HT:PC70BM (1:1)/Si	M4
		glass	P3HT:PC70BM (1:1)/glass	G4
		ITO *	P3HT:PC70BM (1:1)/ITO	I4
		ITO nano #	P3HT:PC70BM (1:1)/Micro-patterned ITO	I4nano
5	Drop-cast	silicon	P3HT:PC70BM (0.7:1)/Si	D5
	MAPLE	silicon	P3HT:PC70BM (0.7:1)/Si	M5
		glass	P3HT:PC70BM (0.7:1)/glass	G5
		ITO *	P3HT:PC70BM (0.7:1)/ITO	I5
		ITO nano #	P3HT:PC70BM (0.7:1)/Micro-patterned ITO	I5nano
6 (with DIO)	Drop-cast	silicon	P3HT:PC70BM (0.7:1)/Si	D6
	MAPLE	silicon	P3HT:PC70BM (0.7:1)/Si	M6
		glass	P3HT:PC70BM (0.7:1)/glass	G6
		ITO *	P3HT:PC70BM (0.7:1)/ITO	I6
		ITO nano #	P3HT:PC70BM (0.7:1)/Micro-patterned ITO	I6nano

* ITO is the name of the glass substrate/primer/resist/ITO film. # ITO nano is the name of the glass substrate/primer/nanoimprinted resist/ITO film.

**Table 3:** Thickness of the organic layers deposited by MAPLE.

Sample	Organic Layer	Thickness (nm)
Reference samples	P3HT	831 ± 39
	P3HT(DIO)	456 ± 30
	PC70BM	947 ± 16
	PC70BM(DIO)	1110 ± 14
I1	P3HT:PC70BM (1:0.7)	381 ± 17
I1nano		471 ± 9
I2	P3HT:PC70BM (1:0.7) with DIO	325 ± 11
I2nano		154 ± 4
I3	P3HT:PC70BM (1:1)	307 ± 27
I3nano		194 ± 9
I4	P3HT:PC70BM (1:1) with DIO	212 ± 11
I4nano		291 ± 23
I5	P3HT:PC70BM (0.7:1)	372 ± 9
I5nano		255 ± 12
I6	P3HT:PC70BM (0.7:1) with DIO	226 ± 21
I6nano		132 ± 11

**Table 4:** Surface amplitude parameters (RMS and Ra) evaluated from the AFM analysis.

Sample	RMS (nm)	Ra (nm)
P3HT	112	84
P3HT(DIO)	105	70
PC70BM	49	39
PC70BM(DIO)	44	36
ITO	1.5	1
ITOnano	140	117
I2	85	55
I2nano	231	190
I3	190	143
I3nano	260	206

## Data Availability

Not applicable.

## References

[1] Shen J.J. (2021). Recently-explored top electrode materials for transparent organic solar cells. Synth. Met..

[2] Green M., Dunlop E., Hohl-Ebinger J., Yoshita M., Kopidakis N., Hao X. (2021). Solar cell efficiency tables (version 57). Prog. Photovoltaics Res. Appl..

[3] Frisk C., Platzer-Björkman C., Olsson J., Szaniawski P., Wätjen J.T., Fjällström V., Salomé P., Edoff M. (2014). Optimizing Ga-profiles for highly efficient Cu(In, Ga)Se2 thin film solar cells in simple and complex defect models. J. Phys. D. Appl. Phys..

[4] Furue S., Ishizuka S., Yamada A., Iioka M., Higuchi H., Shibata H., Niki S. (2013). Cu(In,Ga)Se2 solar cells and mini-modules fabricated on thin soda-lime glass substrates. Sol. Energy Mater. Sol. Cells.

[5] Hosseini T., Flores-Vivian I., Sobolev K., Kouklin N. (2013). Concrete embedded dye-synthesized photovoltaic solar cell. Sci. Rep..

[6] Bett A.W., Dimroth F., Stollwerck G., Sulima O.V. (1999). III-V compounds for solar cell applications. Appl. Phys. A Mater. Sci. Process..

[7] Holliday S., Ashraf R.S., Wadsworth A., Baran D., Yousaf S.A., Nielsen C.B., Tan C.H., Dimitrov S.D., Shang Z., Gasparini N. (2016). High-efficiency and air-stable P3HT-based polymer solar cells with a new non-fullerene acceptor. Nat. Commun..

[8] Usmani B., Ranjan R., Prateek, Gupta S.K., Gupta R.K., Nalwa K.S., Garg A. (2021). Inverted PTB7-Th:PC71BM organic solar cells with 11.8% PCE via incorporation of gold nanoparticles in ZnO electron transport layer. Sol. Energy.

[9] Uddin S.I., Tahir M., Aziz F., Sarker M.R., Muhammad F., Khan D.N., Md Ali S.H. (2020). Thickness optimization and photovoltaic properties of bulk heterojunction solar cells based on pfb–pcbm layer. Energies.

[10] Wang J., Zardetto V., Datta K., Zhang D., Wienk M.M., Janssen R.A.J. (2020). 16.8% Monolithic all-perovskite triple-junction solar cells via a universal two-step solution process. Nat. Commun..

[11] Zheng L., Xuan Y., Wang J., Bao S., Liu X., Zhang K. (2022). Inverted perovskite/silicon V-shaped tandem solar cells with 27.6% efficiency via self-assembled monolayer-modified nickel oxide layer. J. Mater. Chem. A.

[12] Tahir M., Din I.U., Zeb M., Aziz F., Wahab F., Gul Z., Alamgeer, Sarker M.R., Ali S., Ali S.H.M. (2022). Thin Films Characterization and Study of N749-Black Dye for Photovoltaic Applications. Coatings.

[13] Wang D., Liu H., Li Y., Zhou G., Zhan L., Zhu H., Lu X., Chen H., Li C.Z. (2021). High-performance and eco-friendly semitransparent organic solar cells for greenhouse applications. Joule.

[14] Güler E.N., Distler A., Basu R., Brabec C.J. (2022). Fully solution-processed, light-weight, and ultraflexible organic solar cells. Flex. Print. Electron..

[15] Colsmann A., Röhm H., Sprau C. (2020). Shining Light on Organic Solar Cells. Sol. RRL.

[16] Berny S., Blouin N., Distler A., Egelhaaf H.J., Krompiec M., Lohr A., Lozman O.R., Morse G.E., Nanson L., Pron A. (2015). Solar trees: First large-scale demonstration of fully solution coated, semitransparent, flexible organic photovoltaic modules. Adv. Sci..

[17] Sun C., Xia R., Shi H., Yao H., Liu X., Hou J., Huang F., Yip H.L., Cao Y. (2018). Heat-Insulating Multifunctional Semitransparent Polymer Solar Cells. Joule.

[18] Shi H., Xia R., Zhang G., Yip H.L., Cao Y. (2019). Spectral Engineering of Semitransparent Polymer Solar Cells for Greenhouse Applications. Adv. Energy Mater..

[19] Needell D.R., Phelan M.E., Hartlove J.T., Atwater H.A. (2021). Solar power windows: Connecting scientific advances to market signals. Energy.

[20] Glogic E., Weyand S., Tsang M.P., Young S.B., Schebek L., Sonnemann G. (2019). Life cycle assessment of organic photovoltaic charger use in Europe: The role of product use intensity and irradiation. J. Clean. Prod..

[21] Gao Y., Dong J., Isabella O., Santbergen R., Tan H., Zeman M., Zhang G. (2018). A photovoltaic window with sun-tracking shading elements towards maximum power generation and non-glare daylighting. Appl. Energy.

[22] Duvva N., Raptis D., Kumar C.V., Koukaras E.N., Giribabu L., Lianos P. (2016). Design of diketopyrrolopyrrole chromophores applicable as sensitizers in dye-sensitized photovoltaic windows for green houses. Dye. Pigment..

[23] Landerer D., Mertens A., Freis D., Droll R., Leonhard T., Schulz A.D., Bahro D., Colsmann A. (2017). Enhanced thermal stability of organic solar cells comprising ternary D-D-A bulk-heterojunctions. npj Flex. Electron..

[24] Jiang Q., Xing Y. (2020). Improved performance of small molecule organic solar cells by incorporation of a glancing angle deposited donor layer. Sci. Rep..

[25] Xie R., Ishijima N., Sugime H., Noda S. (2019). Enhancing the photovoltaic performance of hybrid heterojunction solar cells by passivation of silicon surface via a simple 1-min annealing process. Sci. Rep..

[26] Shalev G., Schmitt S.W., Embrechts H., Brönstrup G., Christiansen S. (2015). Enhanced photovoltaics inspired by the fovea centralis. Sci. Rep..

[27] Liu Q., Jiang Y., Jin K., Qin J., Xu J., Li W., Xiong J., Liu J., Xiao Z., Sun K. (2020). 18% Efficiency organic solar cells. Sci. Bull..

[28] Liu F., Zhou L., Liu W., Zhou Z., Yue Q., Zheng W., Sun R., Liu W., Xu S., Fan H. (2021). Organic Solar Cells with 18% Efficiency Enabled by an Alloy Acceptor: A Two-in-One Strategy. Adv. Mater..

[29] Weng K., Ye L., Zhu L., Xu J., Zhou J., Feng X., Lu G., Tan S., Liu F., Sun Y. (2020). Optimized active layer morphology toward efficient and polymer batch insensitive organic solar cells. Nat. Commun..

[30] Yun M.J., Sim Y.H., Cha S.I., Seo S.H., Lee D.Y. (2017). High Energy Conversion Efficiency with 3-D Micro-Patterned Photoanode for Enhancement Diffusivity and Modification of Photon Distribution in Dye-Sensitized Solar Cells. Sci. Rep..

[31] Li C., Gu X., Chen Z., Han X., Yu N., Wei Y., Gao J., Chen H., Zhang M., Wang A. (2022). Achieving Record-Efficiency Organic Solar Cells upon Tuning the Conformation of Solid Additives. J. Am. Chem. Soc..

[32] Xie Y., Ryu H.S., Han L., Cai Y., Duan X., Wei D., Woo H.Y., Sun Y. (2021). High-efficiency organic solar cells enabled by an alcohol-washable solid additive. Sci. China Chem..

[33] Bernède J.C., Cattin L., Morsli M., Kanth S.R.B., Patil S., Stephant N. (2012). Improvement of the efficiency of organic solar cells using the terthiophene-pyran-malononitrile (T3PM) as electron donor, through the use of a MoO3/CuI anode buffer layer. Energy Procedia.

[34] Lu H., Liu J., Liu Y., Xu X., Bo Z. (2021). Improving the Efficiency of Organic Solar Cells by Introducing Perylene Diimide Derivative as Third Component and Individually Dissolving Donor/Acceptor. ChemSusChem.

[35] Choi J.H., Choi H.J., Shin J.H., Kim H.P., Jang J., Lee H. (2013). Enhancement of organic solar cell efficiency by patterning the PEDOT:PSS hole transport layer using nanoimprint lithography. Org. Electron..

[36] Yu H., Li Y., Dong Y., Huang X. (2016). Fabrication and Optimization of Polymer Solar Cells Based on P3HT:PC70BM System. Int. J. Photoenergy.

[37] Lee S., Kong J., Lee K. (2016). Air-Stable Organic Solar Cells Using an Iodine-Free Solvent Additive. Adv. Energy Mater..

[38] Wang T., Scarratt N.W., Yi H., Dunbar A.D.F., Pearson A.J., Watters D.C., Glen T.S., Brook A.C., Kingsley J., Buckley A.R. (2013). Fabricating high performance, donor-acceptor copolymer solar cells by spray-coating in air. Adv. Energy Mater..

[39] Vohra V., Razali N.T., Wahi R., Ganzer L., Virgili T. (2022). A comparative study of low-cost coating processes for green & sustainable organic solar cell active layer manufacturing. Opt. Mater. X.

[40] Zhang L., Zhao H., Yuan J., Lin B., Xing Z., Meng X., Ke L., Hu X., Ma W., Yuan Y. (2020). Blade-coated efficient and stable large-area organic solar cells with optimized additive. Org. Electron..

[41] Amruth C., Dubey D.K., Pahlevani M., Welch G.C. (2021). Slot-Die Coating of All Organic/Polymer Layers for Large-Area Flexible OLEDs: Improved Device Performance with Interlayer Modification. Adv. Mater. Technol..

[42] Wienhold K.S., Körstgens V., Grott S., Jiang X., Schwartzkopf M., Roth S.V., Müller-Buschbaum P. (2019). Effect of Solvent Additives on the Morphology and Device Performance of Printed Nonfullerene Acceptor Based Organic Solar Cells. ACS Appl. Mater. Interfaces.

[43] Caricato A.P., Cesaria M., Gigli G., Loiudice A., Luches A., Martino M., Resta V., Rizzo A., Taurino A. (2012). Poly-(3-hexylthiophene)/[6,6]-phenyl-C 61-butyric-acid-methyl- ester bilayer deposition by matrix-assisted pulsed laser evaporation for organic photovoltaic applications. Appl. Phys. Lett..

[44] Caricato A.P., Castillrjo M., Ossi P., Zhigilei L. (2014). MAPLE and MALDI: Theory and Experiments. Lasers in Materials Science.

[45] Socol M., Preda N., Socol G. (2021). Organic thin films deposited by matrix-assisted pulsed laser evaporation (MAPLE) for photovoltaic cell applications: A review. Coatings.

[46] Paquin F., Rivnay J., Salleo A., Stingelin N., Silva C. (2015). Multi-phase semicrystalline microstructures drive exciton dissociation in neat plastic semiconductors. J. Mater. Chem. C.

[47] Gu Y., Wang C., Russell T.P. (2012). Multi-length-scale morphologies in PCPDTBT/PCBM bulk-heterojunction solar cells. Adv. Energy Mater..

[48] McDowell C., Abdelsamie M., Toney M.F., Bazan G.C. (2018). Solvent Additives: Key Morphology-Directing Agents for Solution-Processed Organic Solar Cells. Adv. Mater..

[49] Chang S.Y., Liao H.C., Shao Y.T., Sung Y.M., Hsu S.H., Ho C.C., Su W.F., Chen Y.F. (2013). Enhancing the efficiency of low bandgap conducting polymer bulk heterojunction solar cells using P3HT as a morphology control agent. J. Mater. Chem. A.

[50] Zhao J., Li Y., Yang G., Jiang K., Lin H., Ade H., Ma W., Yan H. (2016). Efficient organic solar cells processed from hydrocarbon solvents. Nat. Energy.

[51] Kwon S., Kang H., Lee J.H., Lee J., Hong S., Kim H., Lee K. (2017). Effect of Processing Additives on Organic Photovoltaics: Recent Progress and Future Prospects. Adv. Energy Mater..

[52] Fontana M.T., Kang H., Yee P.Y., Fan Z., Hawks S.A., Schelhas L.T., Subramaniyan S., Hwang Y.J., Jenekhe S.A., Tolbert S.H. (2018). Low-Vapor-Pressure Solvent Additives Function as Polymer Swelling Agents in Bulk Heterojunction Organic Photovoltaics. J. Phys. Chem. C.

[53] Otieno F., Kotane L., Airo M., Erasmus R.M., Billing C., Wamwangi D., Billing D.G. (2021). Comparative Investigation of Fullerene PC71BM and Non-fullerene ITIC-Th Acceptors Blended With P3HT or PBDB-T Donor Polymers for PV Applications. Front. Energy Res..

[54] Collins B.A., Li Z., Tumbleston J.R., Gann E., Mcneill C.R., Ade H. (2013). Absolute measurement of domain composition and nanoscale size distribution explains performance in PTB7:PC71bm solar cells. Adv. Energy Mater..

[55] Zang Y., Xin Q., Zhao J., Lin J. (2018). Effect of Active Layer Thickness on the Performance of Polymer Solar Cells Based on a Highly Efficient Donor Material of PTB7-Th. J. Phys. Chem. C.

[56] Liu Y., Kirsch C., Gadisa A., Aryal M., Mitran S., Samulski E.T., Lopez R. (2012). Effects of nano-patterned versus simple flat active layers in upright organic photovoltaic devices. J. Phys. D. Appl. Phys..

[57] Phengdaam A., Nootchanat S., Ishikawa R., Lertvachirapaiboon C., Shinbo K., Kato K., Ekgasit S., Baba A. (2021). Improvement of organic solar cell performance by multiple plasmonic excitations using mixed-silver nanoprisms. J. Sci. Adv. Mater. Devices.

[58] Zhang Y.X., Fang J., Li W., Shen Y., De Chen J., Li Y., Gu H., Pelivani S., Zhang M., Li Y. (2019). Synergetic Transparent Electrode Architecture for Efficient Non-Fullerene Flexible Organic Solar Cells with >12% Efficiency. ACS Nano.

[59] Suleimanov S.K., Berger P., Dyskin V.G., Dzhanklich M.U., Bugakov A.G., Dudko O.A., Kulagina N.A., Kim M. (2016). Antireflection composite coatings for organic solar cells. Appl. Sol. Energy (English Transl. Geliotekhnika).

[60] Thanner C., Eibelhuber M. (2021). UV nanoimprint lithography: Geometrical impact on filling properties of nanoscale patterns. Nanomaterials.

[61] Mao H., Zhang L., Wen L., Huang L., Tan L., Chen Y. (2022). Nanoimprint Lithography-Dependent Vertical Composition Gradient in Pseudo-Planar Heterojunction Organic Solar Cells Combined with Sequential Deposition. Adv. Funct. Mater..

[62] He X., Gao F., Tu G., Hasko D.G., Hüttner S., Greenham N.C., Steiner U., Friend R.H., Huck W.T.S. (2011). Formation of well-ordered heterojunctions in polymer:PCBM photovoltaic devices. Adv. Funct. Mater..

[63] Kang M.G., Kim M.S., Kim J., Guo L.J. (2008). Organic solar cells using nanoimprinted transparent metal electrodes. Adv. Mater..

[64] Isegawa T., Okamoto T., Kondo M., Katsumata S., Kubo W. (2019). P3HT:PC61BM solar cell embedding silver nanostripes for light absorption enhancement. Opt. Commun..

[65] Călugăr A.I.R., Antohe V.A., Iftimie S., Radu A., Filipescu M., Ion L., Dinescu M., Antohe Ş. (2020). On the physical and photo-electrical properties of organic photovoltaic cells based on 1,10-Phenanthroline and 5,10,15,20-Tetra(4-pyridyl)-21H,23H-porphine non-fullerene thin films. Appl. Surf. Sci..

[66] Berger P.R., Kim M. (2018). Polymer solar cells: P3HT:PCBM and beyond. J. Renew. Sustain. Energy.

[67] He Y., Li Y. (2011). Fullerene derivative acceptors for high performance polymer solar cells. Phys. Chem. Chem. Phys..

[68] Fan X., Zhao S.L., Chen Y., Zhang J., Yang Q.Q., Gong W., Yuan M.Y., Xu Z., Xu X.R. (2015). Nano structure evolution in P3HT:PC61BM blend films due to the effects of thermal annealing or by adding solvent. Chinese Phys. B.

[69] Xu B., Sai-Anand G., Unni G.E., Jeong H.M., Kim J.S., Kim S.W., Kwon J.B., Bae J.H., Kang S.W. (2019). Pyridine-based additive optimized P3HT:PC61BM nanomorphology for improved performance and stability in polymer solar cells. Appl. Surf. Sci..

[70] Zhang F., Zhuo Z., Zhang J., Wang X., Xu X., Wang Z., Xin Y., Wang J., Wang J., Tang W. (2012). Influence of PC60BM or PC70BM as electron acceptor on the performance of polymer solar cells. Sol. Energy Mater. Sol. Cells.

[71] Socol M., Preda N., Petre G., Costas A., Rasoga O., Popescu-Pelin G., Mihailescu A., Stanculescu A., Socol G. (2020). MAPLE Deposition of Binary and Ternary Organic Bulk Heterojunctions Based on Zinc Phthalocyanine. Coatings.

[72] Nogimura A., Akaike K., Nakanishi R., Eguchi R., Kanai K. (2013). Electronic structure and surface morphology of [6,6]-phenyl-C 71-butyric acid methyl ester films. Org. Electron..

[73] Yu Y.Y., Shih K.Y., Peng Y.C., Chiu Y.C., Kuo C.C., Yang C.C., Chen C.P. (2022). High-efficiency organic photovoltaic cells processed using a non-halogen solvent. Mater. Chem. Phys..

[74] Rasoga O., Thanner C., Semenova O., Avram A.M., Jinga L. Wafer-level fabrication of nanocones structures by UV-nanoimprint and cryogenic deep reactive ion process. Proceedings of the 2021 International Semiconductor Conference (CAS).

[75] Socol M., Preda N., Rasoga O., Costas A., Stanculescu A., Breazu C., Gherendi F., Socol G. (2019). Pulsed laser deposition of indium tin oxide thin films on nanopatterned glass substrates. Coatings.

[76] Breazu C., Socol M., Preda N., Rasoga O., Costas A., Socol G., Petre G., Stanculescu A. (2021). Nucleobases thin films deposited on nanostructured transparent conductive electrodes for optoelectronic applications. Sci. Rep..

[77] Mkawi E.M., Al-Hadeethi Y., Bazuhair R.S., Yousef A.S., Shalaan E., Arkook B., Abdeldaiem A.M., Almalki R., Bekyarova E. (2021). Optimization of Sb2S3 nanocrystal concentrations in P3HT: PCBM layers to improve the performance of polymer solar cells. Polymers.

[78] Brambilla L., Tommasini M., Botiz I., Rahimi K., Agumba J.O., Stingelin N., Zerbi G. (2014). Regio-regular oligo and poly(3-hexyl thiophene): Precise structural markers from the vibrational spectra of oligomer single crystals. Macromolecules.

[79] Brambilla L., Capel Ferrón C., Tommasini M., Hong K., López Navarrete J.T., Hernández V., Zerbi G. (2018). Infrared and multi-wavelength Raman spectroscopy of regio-regular P3HT and its deutero derivatives. J. Raman Spectrosc..

[80] Blazinic V., Ericsson L.K.E., Muntean S.A., Moons E. (2018). Photo-degradation in air of spin-coated PC60BM and PC70BM films. Synth. Met..

[81] Yue G.T., Wu J.H., Xiao Y.M., Ye H.F., Lin J.M., Huang M.L. (2011). Flexible dye-sensitized solar cell based on PCBM/P3HT heterojunction. Chinese Sci. Bull..

[82] Kalonga G. (2013). Characterization and optimization of poly (3-hexylthiophene-2, 5- diyl) (P3HT) and [6, 6] phenyl-C61-butyric acid methyl ester (PCBM) blends for optical absorption. J. Chem. Eng. Mater. Sci..

[83] Socol M., Preda N., Stanculescu A., Breazu C., Florica C., Stanculescu F., Iftimie S., Girtan M., Popescu-Pelin G., Socol G. (2017). Organic heterostructures deposited by MAPLE on AZO substrate. Appl. Surf. Sci..

[84] Shrotriya V., Ouyang J., Tseng R.J., Li G., Yang Y. (2005). Absorption spectra modification in poly(3-hexylthiophene):methanofullerene blend thin films. Chem. Phys. Lett..

[85] Molefe F.V., Khenfouch M., Dhlamini M.S., Mothudi B.M. (2017). Spectroscopic investigation of charge and energy transfer in P3HT/GO nanocomposite for solar cell applications. Adv. Mater. Lett..

[86] Tlahuice-Flores A., Mejia-Rosales S. (2011). Structural and Vibrational Study of PCBM. J. Chem. Chem. Eng..

[87] Wang C., Gann E., Chesman A.S.R., McNeill C.R. (2019). Residual solvent additive enables the nanostructuring of PTB7-Th:PC71BM solar cells via soft lithography. AIP Adv..

[88] Kim J.Y., Lee K., Coates N.E., Moses D., Nguyen T., Dante M., Heeger A.J. (2007). Fabricated by All-Solution Processing. Science (80-. )..

[89] Supriyanto A., Mustaqim A., Agustin M., Ramelan A.H., Suyitno, Rosa E.S., Yofentina, Nurosyid F. (2016). Fabrication of organic solar cells with design blend P3HT: PCBM variation of mass ratio. IOP Conf. Ser. Mater. Sci. Eng..

[90] Siddiqui H., Parra M.R., Pandey P., Qureshi M.S., Haque F.Z. (2019). Combined parametric optimization of P3HT: PC70BM films for efficient bulk-heterojunction solar cells. J. Solid State Electrochem..

[91] Sibley S.P., Argentine S.M., Francis A.H. (1992). A photoluminescence study of C60 and C70. Chem. Phys. Lett..

[92] Zakhidov E., Imomov M., Quvondikov V., Nematov S., Tajibaev I., Saparbaev A., Ismail I., Shahid B., Yang R. (2019). Comparative study of absorption and photoluminescent properties of organic solar cells based on P3HT:PCBM and P3HT:ITIC blends. Appl. Phys. A Mater. Sci. Process..

[93] Griffin J., Pearson A.J., Scarratt N.W., Wang T., Dunbar A.D.F., Yi H., Iraqi A., Buckley A.R., Lidzey D.G. (2015). Organic photovoltaic devices with enhanced efficiency processed from non-halogenated binary solvent blends. Org. Electron..

